# Aqua­chlorido{1-[1-(4-hydroxy­phen­yl)-1*H*-tetra­zol-5-ylsulfan­yl]acetato}(methanol)(1,10-phenanthroline)manganese(II)

**DOI:** 10.1107/S1600536808038245

**Published:** 2008-11-22

**Authors:** Jian-Ling Yin, Yun-Long Feng

**Affiliations:** a Zhejiang Key Laboratory for Reactive Chemistry on Solid Surfaces, Institute of Physical Chemistry, Zhejiang Normal University, Jinhua, Zhejiang 321004, People’s Republic of China

## Abstract

The title complex, [Mn(C_9_H_7_N_4_O_3_S)Cl(C_12_H_8_N_2_)(CH_4_O)(H_2_O)], contains an Mn^II^ ion six-coordinated by one O atom from the 2-[1-(4-hydroxy­phen­yl)-1*H*-tetra­zol-5-ylsulfan­yl]­acetate ligand, two N atoms from a chelating 1,10-phenanthroline ligand, one O atom from a methanol mol­ecule, one Cl atom and one water mol­ecule in a distorted octa­hedral coordination geometry. The existence of O—H⋯Cl, O—H⋯N and O—H⋯O hydrogen bonds further produces a two-dimensional structure.

## Related literature

For general background, see: Hu *et al.* (2006 ▶); Zhang *et al.* (2006 ▶). 
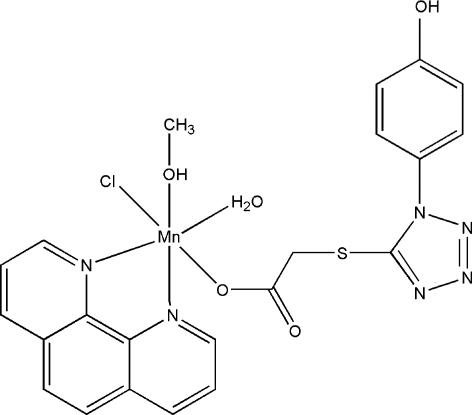


## Experimental

### 

#### Crystal data

[Mn(C_9_H_7_N_4_O_3_S)Cl(C_12_H_8_N_2_)(CH_4_O)(H_2_O)]
*M* *_r_* = 571.90
Triclinic, 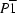

*a* = 10.5565 (3) Å
*b* = 11.4969 (2) Å
*c* = 11.5931 (2) Å
α = 114.362 (1)°
β = 96.841 (1)°
γ = 103.969 (1)°
*V* = 1205.17 (5) Å^3^
*Z* = 2
Mo *K*α radiation
μ = 0.79 mm^−1^
*T* = 296 (2) K
0.34 × 0.23 × 0.11 mm

#### Data collection

Bruker APEXII diffractometer
Absorption correction: multi-scan (*SADABS*; Sheldrick, 1996 ▶) *T*_min_ = 0.806, *T*_max_ = 0.916
13742 measured reflections
4186 independent reflections
3802 reflections with *I* > 2σ(*I*)
*R*_int_ = 0.019

#### Refinement

*R*[*F* ^2^ > 2σ(*F* ^2^)] = 0.027
*wR*(*F* ^2^) = 0.081
*S* = 1.07
4186 reflections
337 parameters
8 restraints
H atoms treated by a mixture of independent and constrained refinement
Δρ_max_ = 0.33 e Å^−3^
Δρ_min_ = −0.27 e Å^−3^

### 

Data collection: *APEX2* (Bruker, 2004 ▶); cell refinement: *SAINT* (Bruker, 2004 ▶); data reduction: *SAINT*; program(s) used to solve structure: *SHELXS97* (Sheldrick, 2008 ▶); program(s) used to refine structure: *SHELXL97* (Sheldrick, 2008 ▶); molecular graphics: *SHELXTL* (Sheldrick, 2008 ▶); software used to prepare material for publication: *SHELXL97*.

## Supplementary Material

Crystal structure: contains datablocks I, global. DOI: 10.1107/S1600536808038245/at2671sup1.cif

Structure factors: contains datablocks I. DOI: 10.1107/S1600536808038245/at2671Isup2.hkl

Additional supplementary materials:  crystallographic information; 3D view; checkCIF report

## Figures and Tables

**Table 1:** Hydrogen-bond geometry (Å, °)

*D*—H⋯*A*	*D*—H	H⋯*A*	*D*⋯*A*	*D*—H⋯*A*
O3—H3*B*⋯Cl1^i^	0.805 (16)	2.334 (17)	3.1344 (16)	173 (2)
O4—H4*B*⋯O1^ii^	0.797 (16)	1.889 (17)	2.6788 (18)	171 (2)
O1*W*—H1*WA*⋯N4^iii^	0.804 (15)	2.006 (15)	2.808 (2)	175 (2)
O1*W*—H1*WB*⋯Cl1^iii^	0.815 (15)	2.376 (17)	3.1665 (15)	164 (2)

Symmetry codes: (i) 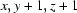; (ii) 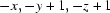; (iii) 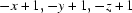.

**Fig. 1.**
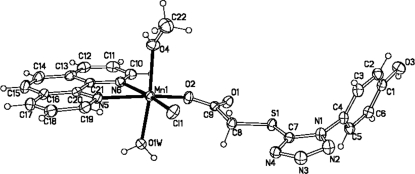
A view of the molecule of (I), showing the atom-labelling scheme. Displacement ellipsoids are shown at the 30% probability level.

## supplementary crystallographic information

### Comment

Recently much interest has been focused on the design and synthesis of complexes based on rigid aromatic carboxylic acids (Hu *et al.*, 2006; Zhang *et al.*, 2006). However, the coordination chemistry and structural properties of complexes based on flexible heterocyclic acetate ligand have been rarely documented to date. 1-(4-hydroxyphenyl)-5-thioacetatetetrazole possesses one phenolic hydroxy group and one flexible thioacetate group, which has senven potential coordinated sites. As illustrated in Fig. 1, Mn^II^ ion is in a distorted octahedral coordination environment, coordinated by one O atom of 1-(4-hydroxyphenyl)-5-thioacetatetetrazole ligand, two N atoms from phen, one O atom from methanol molecule, one Cl atom and one water molecule.

The existence of O—H···Cl, O—H···N, and O—H···O three types of hydrogen bonds further produce two-dimensional structure.

### Experimental

Manganese chloride tetrahydrate 0.5 mmol (0.099 g), 1-(4-hydroxyphenyl)-5-thioacetatetetrazole 0.5 mmol (0.061 g), 1,10-phenanthroline 0.25 mmol (0.050 g), were mixed in 16 ml of 15:1 distilled water/methanol, and stirred for 2 h under 333 K. Then the reaction mixture was filtered and well shaped colourless crystals of the title compound, Mn(H_2_O)Cl(MeOH)(phen)(C_9_H_7_N_4_O_3_S), was obtained from the mother liquor by slow evaporation at room temperature for several days.

### Refinement

The H atoms bonded to C atoms were positioned geometrically [aromatic C—H = 0.93 Å, aliphatic C—H = 0.97 Å, *U*_iso_(H) = 1.2*U*_eq_(C)], and methyl group C—H = 0.96 Å, *U*_iso_(H) = 1.5*U*_eq_(C). The H atoms bonded to O atoms were located in a difference Fourier maps and refined with O—H distance restraints of 0.82 and *U*_iso_(H) = 1.2*U*_eq_(O).

### Crystal data

**Table:**

[Mn(C_9_H_7_N_4_O_3_S)Cl(C_12_H_8_N_2_)(CH_4_O)(H_2_O)]	*Z* = 2
*M**_r_* = 571.90	*F*_000_ = 586
Triclinic, *P*1	*D*_x_ = 1.576 Mg m^−^^3^
Hall symbol: -P 1	Mo *K*α radiation λ = 0.71073 Å
*a* = 10.5565 (3) Å	Cell parameters from 5369 reflections
*b* = 11.4969 (2) Å	θ = 2.0–25.0º
*c* = 11.5931 (2) Å	µ = 0.79 mm^−^^1^
α = 114.3620 (10)º	*T* = 296 (2) K
β = 96.8410 (10)º	Block, colourless
γ = 103.9690 (10)º	0.34 × 0.23 × 0.11 mm
*V* = 1205.17 (5) Å^3^	

### Data collection

**Table:**

Bruker APEXII area-detector diffractometer	4186 independent reflections
Radiation source: fine-focus sealed tube	3802 reflections with *I* > 2σ(*I*)
Monochromator: graphite	*R*_int_ = 0.019
*T* = 296(2) K	θ_max_ = 25.0º
ω scans	θ_min_ = 2.0º
Absorption correction: multi-scan(SADABS; Sheldrick, 1996)	*h* = −12→12
*T*_min_ = 0.806, *T*_max_ = 0.916	*k* = −13→13
13742 measured reflections	*l* = −13→13

### Refinement

**Table:**

Refinement on *F*^2^	Secondary atom site location: difference Fourier map
Least-squares matrix: full	Hydrogen site location: inferred from neighbouring sites
*R*[*F*^2^ > 2σ(*F*^2^)] = 0.027	H atoms treated by a mixture of independent and constrained refinement
*wR*(*F*^2^) = 0.081	*w* = 1/[σ^2^(*F*_o_^2^) + (0.0492*P*)^2^ + 0.2751*P*] where *P* = (*F*_o_^2^ + 2*F*_c_^2^)/3
*S* = 1.07	(Δ/σ)_max_ = 0.001
4186 reflections	Δρ_max_ = 0.33 e Å^−^^3^
337 parameters	Δρ_min_ = −0.27 e Å^−^^3^
8 restraints	Extinction correction: none
Primary atom site location: structure-invariant direct methods	

### Special details

**Table:**

Geometry. All e.s.d.'s (except the e.s.d. in the dihedral angle between two l.s. planes) are estimated using the full covariance matrix. The cell e.s.d.'s are taken into account individually in the estimation of e.s.d.'s in distances, angles and torsion angles; correlations between e.s.d.'s in cell parameters are only used when they are defined by crystal symmetry. An approximate (isotropic) treatment of cell e.s.d.'s is used for estimating e.s.d.'s involving l.s. planes.
Refinement. Refinement of *F*^2^ against ALL reflections. The weighted *R*-factor *wR* and goodness of fit *S* are based on *F*^2^, conventional *R*-factors *R* are based on *F*, with *F* set to zero for negative *F*^2^. The threshold expression of *F*^2^ > σ(*F*^2^) is used only for calculating *R*-factors(gt) *etc*. and is not relevant to the choice of reflections for refinement. *R*-factors based on *F*^2^ are statistically about twice as large as those based on *F*, and *R*- factors based on ALL data will be even larger.

### Fractional atomic coordinates and isotropic or equivalent isotropic displacement parameters (Å^2^)

**Table:**

	*x*	*y*	*z*	*U*_iso_*/*U*_eq_	
Mn1	0.19580 (2)	0.37914 (3)	0.39006 (2)	0.03039 (10)	
S1	0.41984 (5)	0.76432 (5)	0.90045 (5)	0.03782 (13)	
O1	0.22915 (13)	0.76112 (13)	0.71081 (14)	0.0432 (3)	
O2	0.20363 (13)	0.56470 (13)	0.54313 (13)	0.0433 (3)	
O3	0.30543 (16)	1.00580 (15)	1.46112 (16)	0.0506 (4)	
H3B	0.322 (3)	1.0813 (18)	1.469 (3)	0.061*	
O4	0.01155 (13)	0.25511 (15)	0.41066 (15)	0.0459 (3)	
H4B	−0.0559 (19)	0.259 (2)	0.375 (2)	0.055*	
O1W	0.36180 (14)	0.49567 (15)	0.34462 (15)	0.0436 (3)	
H1WA	0.378 (2)	0.461 (2)	0.2748 (16)	0.052*	
H1WB	0.4312 (19)	0.551 (2)	0.3980 (18)	0.052*	
Cl1	0.34450 (5)	0.30009 (5)	0.50090 (5)	0.04618 (14)	
N1	0.55547 (16)	0.73208 (15)	1.09287 (15)	0.0371 (4)	
N2	0.63935 (18)	0.66518 (18)	1.11195 (17)	0.0454 (4)	
N3	0.65952 (17)	0.59784 (17)	0.99986 (17)	0.0443 (4)	
N4	0.59187 (15)	0.61830 (16)	0.90566 (16)	0.0376 (4)	
N5	0.17387 (14)	0.21255 (14)	0.18296 (15)	0.0327 (3)	
N6	0.05314 (15)	0.40551 (15)	0.24743 (15)	0.0331 (3)	
C1	0.37482 (19)	0.94561 (19)	1.37606 (18)	0.0371 (4)	
C2	0.3594 (2)	0.8111 (2)	1.34184 (19)	0.0401 (4)	
H2A	0.3079	0.7679	1.3811	0.048*	
C3	0.4201 (2)	0.74207 (19)	1.25000 (19)	0.0394 (4)	
H3A	0.4083	0.6515	1.2255	0.047*	
C4	0.49903 (19)	0.80822 (18)	1.19405 (18)	0.0352 (4)	
C5	0.5208 (2)	0.94331 (19)	1.23160 (19)	0.0389 (4)	
H5A	0.5768	0.9877	1.1961	0.047*	
C6	0.45784 (19)	1.01185 (19)	1.32297 (19)	0.0382 (4)	
H6A	0.4714	1.1029	1.3489	0.046*	
C7	0.52702 (18)	0.70145 (17)	0.96571 (18)	0.0326 (4)	
C8	0.38078 (19)	0.64340 (19)	0.72948 (18)	0.0371 (4)	
H8A	0.4581	0.6597	0.6940	0.045*	
H8B	0.3587	0.5526	0.7204	0.045*	
C9	0.26138 (17)	0.65837 (18)	0.65563 (18)	0.0325 (4)	
C10	−0.00740 (19)	0.4990 (2)	0.2799 (2)	0.0405 (4)	
H10A	0.0080	0.5585	0.3678	0.049*	
C11	−0.0932 (2)	0.5116 (2)	0.1878 (2)	0.0487 (5)	
H11A	−0.1347	0.5778	0.2144	0.058*	
C12	−0.1158 (2)	0.4263 (2)	0.0587 (2)	0.0497 (5)	
H12A	−0.1732	0.4336	−0.0035	0.060*	
C13	−0.05201 (19)	0.3272 (2)	0.0198 (2)	0.0419 (5)	
C14	−0.0662 (2)	0.2360 (2)	−0.1134 (2)	0.0544 (6)	
H14A	−0.1214	0.2404	−0.1793	0.065*	
C15	−0.0016 (2)	0.1441 (2)	−0.1458 (2)	0.0538 (6)	
H15A	−0.0117	0.0873	−0.2335	0.065*	
C16	0.0824 (2)	0.1320 (2)	−0.04811 (19)	0.0419 (5)	
C17	0.1503 (2)	0.0364 (2)	−0.0762 (2)	0.0495 (5)	
H17A	0.1435	−0.0225	−0.1624	0.059*	
C18	0.2257 (2)	0.0301 (2)	0.0226 (2)	0.0481 (5)	
H18A	0.2708	−0.0332	0.0050	0.058*	
C19	0.23497 (19)	0.11972 (19)	0.1513 (2)	0.0404 (4)	
H19A	0.2867	0.1140	0.2184	0.048*	
C20	0.09807 (17)	0.21888 (18)	0.08438 (18)	0.0322 (4)	
C21	0.03157 (17)	0.32046 (18)	0.11916 (18)	0.0328 (4)	
C22	−0.0099 (3)	0.1695 (3)	0.4730 (3)	0.0630 (6)	
H22A	−0.1049	0.1314	0.4615	0.095*	
H22B	0.0331	0.2215	0.5646	0.095*	
H22C	0.0276	0.0984	0.4346	0.095*	

### Atomic displacement parameters (Å^2^)

**Table:**

	*U*^11^	*U*^22^	*U*^33^	*U*^12^	*U*^13^	*U*^23^
Mn1	0.02964 (16)	0.03098 (16)	0.02770 (17)	0.01230 (12)	0.00583 (12)	0.00973 (13)
S1	0.0417 (3)	0.0368 (3)	0.0306 (3)	0.0196 (2)	0.0053 (2)	0.0085 (2)
O1	0.0404 (7)	0.0346 (7)	0.0434 (8)	0.0181 (6)	0.0034 (6)	0.0060 (6)
O2	0.0435 (8)	0.0426 (8)	0.0310 (7)	0.0208 (6)	0.0029 (6)	0.0030 (6)
O3	0.0562 (9)	0.0466 (9)	0.0550 (10)	0.0260 (8)	0.0250 (8)	0.0208 (8)
O4	0.0320 (7)	0.0576 (9)	0.0558 (10)	0.0166 (7)	0.0104 (7)	0.0320 (8)
O1W	0.0395 (8)	0.0434 (8)	0.0362 (8)	0.0050 (6)	0.0130 (6)	0.0109 (7)
Cl1	0.0447 (3)	0.0375 (3)	0.0495 (3)	0.0167 (2)	−0.0039 (2)	0.0159 (2)
N1	0.0453 (9)	0.0353 (8)	0.0324 (9)	0.0192 (7)	0.0084 (7)	0.0140 (7)
N2	0.0535 (10)	0.0471 (10)	0.0423 (10)	0.0269 (8)	0.0104 (8)	0.0212 (8)
N3	0.0470 (10)	0.0459 (10)	0.0438 (10)	0.0239 (8)	0.0125 (8)	0.0189 (8)
N4	0.0385 (8)	0.0383 (9)	0.0371 (9)	0.0172 (7)	0.0115 (7)	0.0152 (7)
N5	0.0294 (7)	0.0296 (8)	0.0324 (7)	0.0095 (6)	0.0048 (6)	0.0088 (6)
N6	0.0310 (7)	0.0340 (8)	0.0352 (9)	0.0120 (6)	0.0084 (6)	0.0158 (7)
C1	0.0378 (10)	0.0397 (10)	0.0308 (10)	0.0171 (8)	0.0039 (8)	0.0121 (8)
C2	0.0460 (11)	0.0391 (10)	0.0372 (11)	0.0127 (9)	0.0102 (9)	0.0198 (9)
C3	0.0520 (11)	0.0306 (9)	0.0347 (11)	0.0151 (8)	0.0080 (9)	0.0138 (8)
C4	0.0431 (10)	0.0341 (10)	0.0269 (9)	0.0162 (8)	0.0060 (8)	0.0112 (8)
C5	0.0470 (11)	0.0357 (10)	0.0350 (11)	0.0134 (8)	0.0100 (9)	0.0171 (9)
C6	0.0455 (11)	0.0297 (9)	0.0375 (11)	0.0151 (8)	0.0054 (9)	0.0132 (8)
C7	0.0333 (9)	0.0297 (9)	0.0310 (10)	0.0089 (7)	0.0077 (8)	0.0111 (8)
C8	0.0369 (10)	0.0389 (10)	0.0308 (10)	0.0178 (8)	0.0069 (8)	0.0088 (8)
C9	0.0290 (9)	0.0331 (10)	0.0315 (10)	0.0105 (7)	0.0088 (8)	0.0104 (8)
C10	0.0394 (10)	0.0386 (10)	0.0475 (12)	0.0165 (8)	0.0145 (9)	0.0202 (9)
C11	0.0410 (11)	0.0507 (12)	0.0704 (16)	0.0237 (10)	0.0162 (11)	0.0370 (12)
C12	0.0379 (11)	0.0564 (13)	0.0622 (15)	0.0126 (10)	0.0027 (10)	0.0382 (12)
C13	0.0335 (10)	0.0460 (11)	0.0443 (12)	0.0050 (8)	0.0006 (8)	0.0257 (10)
C14	0.0513 (13)	0.0608 (14)	0.0402 (13)	0.0046 (11)	−0.0061 (10)	0.0250 (11)
C15	0.0590 (13)	0.0531 (13)	0.0291 (11)	0.0032 (11)	0.0011 (10)	0.0109 (10)
C16	0.0390 (10)	0.0368 (10)	0.0334 (11)	0.0004 (8)	0.0062 (8)	0.0081 (9)
C17	0.0508 (12)	0.0381 (11)	0.0390 (12)	0.0061 (9)	0.0152 (10)	0.0021 (9)
C18	0.0422 (11)	0.0338 (11)	0.0557 (14)	0.0133 (9)	0.0162 (10)	0.0072 (10)
C19	0.0358 (10)	0.0341 (10)	0.0458 (12)	0.0132 (8)	0.0090 (9)	0.0125 (9)
C20	0.0271 (8)	0.0306 (9)	0.0318 (10)	0.0036 (7)	0.0057 (7)	0.0111 (8)
C21	0.0271 (8)	0.0348 (10)	0.0339 (10)	0.0050 (7)	0.0048 (7)	0.0168 (8)
C22	0.0745 (16)	0.0582 (15)	0.0655 (17)	0.0219 (13)	0.0239 (13)	0.0347 (12)

### Geometric parameters (Å, °)

**Table:**

Mn1—O2	2.1128 (13)	C3—H3A	0.9300
Mn1—O1W	2.1937 (13)	C4—C5	1.379 (3)
Mn1—O4	2.2195 (14)	C5—C6	1.384 (3)
Mn1—N6	2.2659 (15)	C5—H5A	0.9300
Mn1—N5	2.3121 (15)	C6—H6A	0.9300
Mn1—Cl1	2.4725 (5)	C8—C9	1.523 (3)
S1—C7	1.7370 (18)	C8—H8A	0.9700
S1—C8	1.8129 (19)	C8—H8B	0.9700
O1—C9	1.242 (2)	C10—C11	1.395 (3)
O2—C9	1.252 (2)	C10—H10A	0.9300
O3—C1	1.357 (2)	C11—C12	1.360 (3)
O3—H3B	0.805 (16)	C11—H11A	0.9300
O4—C22	1.435 (3)	C12—C13	1.405 (3)
O4—H4B	0.797 (16)	C12—H12A	0.9300
O1W—H1WA	0.804 (15)	C13—C21	1.405 (3)
O1W—H1WB	0.815 (15)	C13—C14	1.429 (3)
N1—C7	1.343 (2)	C14—C15	1.343 (3)
N1—N2	1.359 (2)	C14—H14A	0.9300
N1—C4	1.438 (2)	C15—C16	1.427 (3)
N2—N3	1.284 (2)	C15—H15A	0.9300
N3—N4	1.366 (2)	C16—C17	1.404 (3)
N4—C7	1.322 (2)	C16—C20	1.406 (3)
N5—C19	1.325 (2)	C17—C18	1.353 (3)
N5—C20	1.353 (2)	C17—H17A	0.9300
N6—C10	1.326 (2)	C18—C19	1.395 (3)
N6—C21	1.353 (2)	C18—H18A	0.9300
C1—C6	1.386 (3)	C19—H19A	0.9300
C1—C2	1.390 (3)	C20—C21	1.447 (2)
C2—C3	1.373 (3)	C22—H22A	0.9600
C2—H2A	0.9300	C22—H22B	0.9600
C3—C4	1.387 (3)	C22—H22C	0.9600
			
O2—Mn1—O1W	86.83 (6)	N4—C7—N1	108.36 (16)
O2—Mn1—O4	96.20 (6)	N4—C7—S1	128.91 (15)
O1W—Mn1—O4	172.42 (6)	N1—C7—S1	122.72 (13)
O2—Mn1—N6	90.89 (5)	C9—C8—S1	108.62 (12)
O1W—Mn1—N6	87.38 (6)	C9—C8—H8A	110.0
O4—Mn1—N6	85.63 (5)	S1—C8—H8A	110.0
O2—Mn1—N5	161.07 (6)	C9—C8—H8B	110.0
O1W—Mn1—N5	83.48 (5)	S1—C8—H8B	110.0
O4—Mn1—N5	91.61 (6)	H8A—C8—H8B	108.3
N6—Mn1—N5	72.49 (5)	O1—C9—O2	125.37 (17)
O2—Mn1—Cl1	102.48 (4)	O1—C9—C8	118.10 (16)
O1W—Mn1—Cl1	93.64 (4)	O2—C9—C8	116.53 (15)
O4—Mn1—Cl1	92.49 (4)	N6—C10—C11	122.7 (2)
N6—Mn1—Cl1	166.62 (4)	N6—C10—H10A	118.6
N5—Mn1—Cl1	94.35 (4)	C11—C10—H10A	118.6
C7—S1—C8	99.25 (8)	C12—C11—C10	119.42 (19)
C9—O2—Mn1	148.24 (12)	C12—C11—H11A	120.3
C1—O3—H3B	106.1 (18)	C10—C11—H11A	120.3
C22—O4—Mn1	133.01 (14)	C11—C12—C13	119.71 (19)
C22—O4—H4B	114.4 (17)	C11—C12—H12A	120.1
Mn1—O4—H4B	112.6 (17)	C13—C12—H12A	120.1
Mn1—O1W—H1WA	118.9 (16)	C12—C13—C21	117.14 (19)
Mn1—O1W—H1WB	124.4 (16)	C12—C13—C14	123.71 (19)
H1WA—O1W—H1WB	108.7 (19)	C21—C13—C14	119.14 (19)
C7—N1—N2	108.33 (15)	C15—C14—C13	121.5 (2)
C7—N1—C4	128.64 (15)	C15—C14—H14A	119.3
N2—N1—C4	122.67 (15)	C13—C14—H14A	119.3
N3—N2—N1	106.35 (16)	C14—C15—C16	121.3 (2)
N2—N3—N4	111.19 (15)	C14—C15—H15A	119.4
C7—N4—N3	105.76 (15)	C16—C15—H15A	119.4
C19—N5—C20	117.63 (16)	C17—C16—C20	117.17 (19)
C19—N5—Mn1	126.95 (13)	C17—C16—C15	123.71 (19)
C20—N5—Mn1	115.21 (11)	C20—C16—C15	119.11 (19)
C10—N6—C21	118.22 (16)	C18—C17—C16	119.83 (19)
C10—N6—Mn1	125.14 (13)	C18—C17—H17A	120.1
C21—N6—Mn1	116.62 (11)	C16—C17—H17A	120.1
O3—C1—C6	123.01 (17)	C17—C18—C19	119.14 (19)
O3—C1—C2	117.16 (17)	C17—C18—H18A	120.4
C6—C1—C2	119.83 (17)	C19—C18—H18A	120.4
C3—C2—C1	119.98 (18)	N5—C19—C18	123.4 (2)
C3—C2—H2A	120.0	N5—C19—H19A	118.3
C1—C2—H2A	120.0	C18—C19—H19A	118.3
C2—C3—C4	119.58 (17)	N5—C20—C16	122.84 (17)
C2—C3—H3A	120.2	N5—C20—C21	117.54 (16)
C4—C3—H3A	120.2	C16—C20—C21	119.62 (17)
C5—C4—C3	121.15 (17)	N6—C21—C13	122.75 (17)
C5—C4—N1	120.23 (16)	N6—C21—C20	117.93 (16)
C3—C4—N1	118.59 (16)	C13—C21—C20	119.32 (17)
C4—C5—C6	118.93 (18)	O4—C22—H22A	109.5
C4—C5—H5A	120.5	O4—C22—H22B	109.5
C6—C5—H5A	120.5	H22A—C22—H22B	109.5
C5—C6—C1	120.40 (17)	O4—C22—H22C	109.5
C5—C6—H6A	119.8	H22A—C22—H22C	109.5
C1—C6—H6A	119.8	H22B—C22—H22C	109.5
			
O1W—Mn1—O2—C9	−77.1 (2)	N2—N1—C7—N4	−0.5 (2)
O4—Mn1—O2—C9	109.8 (2)	C4—N1—C7—N4	−173.59 (17)
N6—Mn1—O2—C9	−164.5 (2)	N2—N1—C7—S1	178.75 (13)
N5—Mn1—O2—C9	−136.3 (2)	C4—N1—C7—S1	5.7 (3)
Cl1—Mn1—O2—C9	15.9 (3)	C8—S1—C7—N4	18.08 (19)
O2—Mn1—O4—C22	−99.15 (19)	C8—S1—C7—N1	−161.03 (16)
N6—Mn1—O4—C22	170.4 (2)	C7—S1—C8—C9	166.78 (13)
N5—Mn1—O4—C22	98.12 (19)	Mn1—O2—C9—O1	177.25 (16)
Cl1—Mn1—O4—C22	3.69 (19)	Mn1—O2—C9—C8	−2.8 (3)
C7—N1—N2—N3	0.1 (2)	S1—C8—C9—O1	14.7 (2)
C4—N1—N2—N3	173.67 (17)	S1—C8—C9—O2	−165.34 (14)
N1—N2—N3—N4	0.4 (2)	C21—N6—C10—C11	0.9 (3)
N2—N3—N4—C7	−0.7 (2)	Mn1—N6—C10—C11	179.25 (14)
O2—Mn1—N5—C19	149.03 (17)	N6—C10—C11—C12	−0.7 (3)
O1W—Mn1—N5—C19	89.35 (15)	C10—C11—C12—C13	−0.3 (3)
O4—Mn1—N5—C19	−96.44 (15)	C11—C12—C13—C21	1.0 (3)
N6—Mn1—N5—C19	178.65 (16)	C11—C12—C13—C14	−177.97 (19)
Cl1—Mn1—N5—C19	−3.82 (15)	C12—C13—C14—C15	179.1 (2)
O2—Mn1—N5—C20	−25.6 (2)	C21—C13—C14—C15	0.1 (3)
O1W—Mn1—N5—C20	−85.32 (12)	C13—C14—C15—C16	1.1 (3)
O4—Mn1—N5—C20	88.88 (12)	C14—C15—C16—C17	178.9 (2)
N6—Mn1—N5—C20	3.98 (11)	C14—C15—C16—C20	−0.4 (3)
Cl1—Mn1—N5—C20	−178.50 (11)	C20—C16—C17—C18	0.7 (3)
O2—Mn1—N6—C10	−10.40 (15)	C15—C16—C17—C18	−178.6 (2)
O1W—Mn1—N6—C10	−97.18 (15)	C16—C17—C18—C19	−0.3 (3)
O4—Mn1—N6—C10	85.75 (15)	C20—N5—C19—C18	0.5 (3)
N5—Mn1—N6—C10	178.83 (16)	Mn1—N5—C19—C18	−174.03 (14)
Cl1—Mn1—N6—C10	168.12 (13)	C17—C18—C19—N5	−0.4 (3)
O2—Mn1—N6—C21	167.97 (12)	C19—N5—C20—C16	−0.1 (3)
O1W—Mn1—N6—C21	81.19 (12)	Mn1—N5—C20—C16	175.10 (13)
O4—Mn1—N6—C21	−95.88 (12)	C19—N5—C20—C21	−179.93 (15)
N5—Mn1—N6—C21	−2.80 (11)	Mn1—N5—C20—C21	−4.72 (19)
Cl1—Mn1—N6—C21	−13.5 (3)	C17—C16—C20—N5	−0.5 (3)
O3—C1—C2—C3	175.91 (18)	C15—C16—C20—N5	178.84 (17)
C6—C1—C2—C3	−3.7 (3)	C17—C16—C20—C21	179.34 (17)
C1—C2—C3—C4	1.4 (3)	C15—C16—C20—C21	−1.3 (3)
C2—C3—C4—C5	1.8 (3)	C10—N6—C21—C13	−0.1 (3)
C2—C3—C4—N1	−176.58 (17)	Mn1—N6—C21—C13	−178.56 (13)
C7—N1—C4—C5	−65.4 (3)	C10—N6—C21—C20	179.92 (15)
N2—N1—C4—C5	122.4 (2)	Mn1—N6—C21—C20	1.4 (2)
C7—N1—C4—C3	113.0 (2)	C12—C13—C21—N6	−0.9 (3)
N2—N1—C4—C3	−59.2 (3)	C14—C13—C21—N6	178.17 (17)
C3—C4—C5—C6	−2.6 (3)	C12—C13—C21—C20	179.12 (16)
N1—C4—C5—C6	175.72 (17)	C14—C13—C21—C20	−1.8 (3)
C4—C5—C6—C1	0.3 (3)	N5—C20—C21—N6	2.3 (2)
O3—C1—C6—C5	−176.73 (18)	C16—C20—C21—N6	−177.54 (16)
C2—C1—C6—C5	2.9 (3)	N5—C20—C21—C13	−177.72 (15)
N3—N4—C7—N1	0.7 (2)	C16—C20—C21—C13	2.5 (2)
N3—N4—C7—S1	−178.50 (14)		

### Hydrogen-bond geometry (Å, °)

**Table:**

*D*—H···*A*	*D*—H	H···*A*	*D*···*A*	*D*—H···*A*
O3—H3B···Cl1^i^	0.805 (16)	2.334 (17)	3.1344 (16)	173 (2)
O4—H4B···O1^ii^	0.797 (16)	1.889 (17)	2.6788 (18)	171 (2)
O1W—H1WA···N4^iii^	0.804 (15)	2.006 (15)	2.808 (2)	175 (2)
O1W—H1WB···Cl1^iii^	0.815 (15)	2.376 (17)	3.1665 (15)	164 (2)

Symmetry codes: (i) *x*, *y*+1, *z*+1; (ii) −*x*, −*y*+1, −*z*+1; (iii) −*x*+1, −*y*+1, −*z*+1.
